# Safety management in times of crisis: Lessons learned from a nationwide status-analysis on German intensive care units during the COVID-19 pandemic

**DOI:** 10.3389/fmed.2022.988746

**Published:** 2022-10-05

**Authors:** Michelle Schmidt, Sophie Isabelle Lambert, Martin Klasen, Benedikt Sandmeyer, Marc Lazarovici, Franziska Jahns, Lara Charlott Trefz, Gunther Hempel, Saša Sopka

**Affiliations:** ^1^AIXTRA — Competence Center for Training and Patient Safety, Medical Faculty, RWTH Aachen University, Aachen, Germany; ^2^Department of Anaesthesiology, University Hospital RWTH Aachen, Medical Faculty, RWTH Aachen University, Aachen, Germany; ^3^Institut für Notfallmedizin und Medizinmanagement (INM), Klinikum der Universität München, LMU München, München, Germany; ^4^Department of Anaesthesiology and Intensive Care, University Medical Centre Schleswig-Holstein, Campus Lübeck, Lübeck, Germany; ^5^Department of Anaesthesiology and Intensive Care, University of Leipzig Medical Centre, Leipzig, Germany

**Keywords:** Safety Management, human factor, medical error, patient safety culture, Intensive Care Unit (ICU), COVID-19 pandemic

## Abstract

**Background:**

The status of Safety Management is highly relevant to evaluate an organization's ability to deal with unexpected events or errors, especially in times of crisis. However, it remains unclear to what extent Safety Management was developed and sufficiently implemented within the healthcare system during the COVID-19 pandemic. Providing insights of potential for improvement is expected to be directional for ongoing Safety Management efforts, in times of crisis and beyond.

**Method:**

A nationwide survey study was conducted among healthcare professionals and auxiliary staff on German Intensive Care Units (ICUs) evaluating their experiences during the first wave of the COVID-19 pandemic. Error Management and Patient Safety Culture (PSC) measures served to operationalize Safety Management. Data were analyzed descriptively and by using quantitative content analysis (QCA).

**Results:**

Results for *n* = 588 participants from 53 hospitals show that there is a gap between errors occurred, reported, documented, and addressed. QCA revealed that low quality of safety culture (27.8%) was the most mentioned reason for errors not being addressed. Overall, ratings of PSC ranged from 26.7 to 57.9% positive response with *Staffing* being the worst and *Teamwork Within Units* being the best rated dimension. While assessments showed a similar pattern, medical staff rated PSC on ICUs more positively in comparison to nursing staff.

**Conclusion:**

The status-analysis of Safety Management in times of crisis revealed relevant potential for improvement. Human Factor plays a crucial role in the occurrence and the way errors are dealt with on ICUs, but systemic factors should not be underestimated. Further intensified efforts specifically in the fields of staffing and error reporting, documentation and communication are needed to improve Safety Management on ICUs. These findings might also be applicable across nations and sectors beyond the medical field.

## Introduction

Medical errors are a major threat to patient safety (1). Over the past two years, healthcare systems all over the world are confronted with an outstanding and long-lasting crisis – the COVID-19 pandemic. According to Schilling et al. (2), the first German case of COVID-19 was reported on January 27th, 2020. At the beginning of March 2020, the momentum of the worldwide pandemic spread in Germany. Up until October 2020, 152.984 cases of COVID-19 infections were reported whereof approximately 18% of cases had to be hospitalized. Among all patients who were hospitalized, 14% had to be treated on ICU whereof 23% were depending on ventilation (2). Taking all cases into account, there was a fatal course of disease in 5% of cases. On a national level, there was an excess mortality rate of 5% in comparison to 2019 (3).

It seems more relevant than ever to find out whether existing efforts were able to strengthen the organization to an extent that they are well prepared for a crisis like this. The question arises what the status of Safety Management in the critical starting phase of the crisis was and how healthcare organizations cope with a crisis such as the pandemic. Safety is referred to as the absence of unwanted outcomes such as incidents or accidents whereas the aspect of management includes regulation or control mechanisms (4). Goodman (5) states that Safety Management is related to the culture of an organization and includes organizational as well as behavioral elements of the system and its processes (5, 6). It is a concept that aims to “develop organizational policies and procedures to foster an environment where safety is so highly valued that members practice the highest safety behaviors” (5).

20 years after the institute of medicine claimed medical errors as a major issue causing up to 98.000 deaths per year within the United States (7), much has been researched about efforts on safety management, particularly regarding patient safety culture (PSC) and the detection and processing of medical errors (e.g., (8–10). Preventable adverse events (PAE) are often reported in the context of medical errors as they are defined as injuries due to a non-intercepted serious medical error (11). A systematic review of 151 international studies by the german coalition for patient safety showed PAE account for approximately 17.000 deaths in Germany per year (8). Furthermore, the economic burden is not to be neglected as patient harm causes high financial costs (12). Nevertheless, previous studies criticize existing efforts in improving safety management within the organizations as insufficient (13, 14).

The healthcare system is a complex system where errors can have severe consequences (15). Studies show that a considerable number of these serious medical errors occur in the Intensive Care Units (ICUs), with a common potential for patient harm (11, 16–18). Donchin et al. (16) estimated that 1.7 errors occur in the ICUs per patient and day. The environment of ICUs is characterized by complexity, time pressure and constantly changing conditions where decisions often need to be made without a comprehensive information base. Dealing with technical equipment is a quite common task and expected to further increase, especially when taking into consideration the rise of decision-guiding technology of artificial intelligence. Nevertheless, implementation of increasingly advanced equipment and technology does not completely eliminate medical errors regarding human factors (15). Quite the contrary, ICUs are often described as particularly prone to medical errors (19). Looking at a major event of a crisis such as the COVID-19 pandemic, which particularly affected the ICUs in an outstanding way, this part of the health organization is therefore of special interest to Safety Management efforts.

Looking closer into human errors, Reason (20) proposed two different lenses, 1) the person-approach and 2) the system-approach. The person-approach on the one hand considers human behavior as the cause of errors through unsafe actions arising from mental processes, e. g. forgetting, poor motivation. The system-approach on the other hand focuses on analyzing and changing conditions under which humans work, assuming that human nature itself cannot be changed (20). This assumption is also frequently emphasized by the quote “to err is human” (7). To support this, research shows that situations rather than individuals are error-prone (15). Causes of errors are well explored regarding fatigue, workload as well as burnout (21), therefore, the anticipation is reasonable that medical errors can derive from stress related to pandemic circumstances. Nevertheless, human errors are very unstable, hard to predict and they can only be avoided to a certain extent (15, 19). It is therefore crucial to not simply follow error prevention strategies and try to eradicate human errors because they can as well be developmental and offer enormous potential for organizational learning (15, 22). For the present study we followed the system-approach. Especially in times of a major crisis, the question needs to be raised how system adaptations can increase future organizational learning as well as organizational resilience.

In summary, it remains unclear to what extent past Safety Management efforts in improving PSC or Error Management (EM) have prepared healthcare organizations for an unexpected crisis. What is the perception of EM and PSC when asking workforces in one of the most pressured parts of the organization and at times of unprecedented change? The aim of the present study is to provide an answer to this question and improve the understanding of Safety Management within the interactions between ICUs and a broader disruptive factor of a crisis such as the COVID-19 pandemic. Therefore, we conducted a nationwide survey on German ICUs during the first wave of the COVID-19 pandemic.

## Methods

### Ethics

Ethical approval (Ethics Review Board document 459/20) was granted according to the ethical principles of the World Medical Association's Declaration of Helsinki (23) on 01.12.2020 by the Institutional Ethics Review Board of the University Hospital, RWTH Aachen.

### Study design

We conducted a nationwide cross-sectional survey study addressing the topics of EM and PSC. The present study was based on individual reports of errors that occurred during the first wave of the COVID-19 pandemic. Individual perceptions can vary greatly from objective reportings, whereas we aimed at providing in-depth-analysis of subjective assessments rather than solely looking at quantitative data. Furthermore, studies have shown the inadequacy of voluntary error reportings to draw a complete picture of EM and PSC (18). Moreover, the first wave of the pandemic is of special interest to the present study as healthcare organizations were expected to be at the maximum of organizational change during this phase. As the COVID-19 pandemic started, no organizational learning effects were to be expected at this point of time, the staff was assumed to be in a reaction mode. Additionally, the recall of errors recalling the first wave of the pandemic was expected to be extremely high as the staff was confronted with drastic changes for the first time during the crisis.

### Questionnaire design

Items in the questionnaire were not randomized to allow for coherence when filling out the survey. The questionnaire spread over 15 pages including the first page with an informed consent, an informatory page about the definition of error and a last page acknowledging participation. In total, 70 items were presented to participants on 12 pages. Due to adaptive questioning applying to some items, e. g. when asking for profession-specific further trainings, the number of items presented to each individual participant was lower than 70 and may have varied per participant depending on particular responses. Participants were not able to review and change their answers after continuing to the next screen of the questionnaire.

### Sample selection process

Participants of the study included healthcare professionals as well as auxiliary staff in the fields of medicine and nursing of all hierarchical levels on German ICUs. To avoid sample selection bias and generate a study sample that contains a broad range of hospitals all over Germany, a defined selection process was conducted before participant recruitment. Therefore, we selected hospitals stratified by *geographical distribution, hospital type* as well as *COVID-19 patient load*. In terms of geographical distribution, we selected hospitals out of the north (Schleswig-Holstein, Saxony, Lower Saxony, Mecklenburg-Western Pomerania, Hamburg, Bremen, Brandenburg, Berlin, Saxony-Anhalt), the middle (Thuringia, Northrhine-Westfalia, Hesse) and the south (Baden-Württemberg, Bavaria, Rhineland-Palatinate, Saarland) of Germany equally. Hospital type ensured that university hospitals as well as specialty clinics, municipal hospitals and other hospital types were included. Upon request, the Robert Koch Institute provided us with data about *COVID-19 patient load* for every hospital within the registry of the German Interdisciplinary Association for Intensive Care and Emergency Medicine. These data included information about how many COVID-19 patients have been treated in the respective hospital during the first wave of the COVID-19 pandemic until the cut-off date 30th of July 2020. Hence, a balanced number of hospitals with low (< 20 patients), medium (20–59 patients) and high (> 59 patients) COVID-19 patient load was included in the study sample. Furthermore, solely hospitals that provide ICUs were included in the study sample. This process finally led to a selection of 152 out of 1.200 hospitals.

### Data collection

After careful programming and design of the questionnaire with SoSci Survey (24) and publication *via* www.soscisurvey.de, the questionnaire was tested for coherence, technical functionality as well as time needed for completion by five colleagues by means of a pre-test. Data collection was carried out between April 6th and June 20th, 2021 through an open survey. Participation was voluntary and anonymous. Due to data restriction, neither Cookies nor IP-addresses were recorded. In the informed consent at the beginning of the questionnaire, participants were provided with information about the study and time needed for completion. First, central contact persons were identified for every selected hospital. This was either the chief medical officer (CMO) or a person named by the CMO. This person was then contacted *via* mail and asked to share the survey with ICU staff at their sites. Survey invitation mails and posters with QR-Codes were developed for this purpose and provided to the contact persons. In case of no response, hospitals were contacted again *via* mail or phone call and asked for participation. The survey was reported according to the CHERRIES checklist (25) (Table 1 in Supplementary material).

### Measures

#### Error management

**Figure 1:**
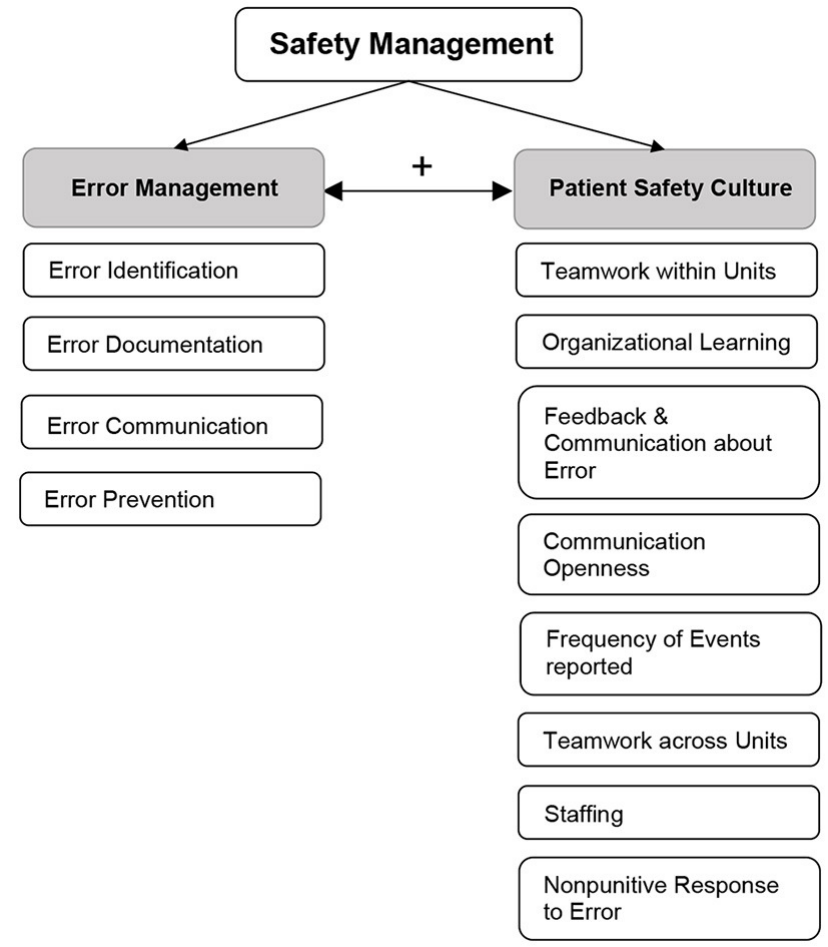
Theoretical framework of Safety Management operationalized by the concepts of EM and PSC.

We propose EM as a concept that serves to operationalize an essential part of Safety Management. We define EM as an overarching construct bringing together an understanding of the nature and extent of errors, conditions that promote the emergence of errors as well as human behavior to prevent, mitigate errors or cope with their consequences (20, 26). We assessed EM in the proposed dimensions of *Error Identification* (How are medical errors detected and who detects them?), *Error Documentation* (How are medical errors documented?), *Error Communication* (How are medical errors addressed within the team?) and *Error Prevention* (How are medical errors prevented from happening again?). The mainly open-ended items were developed in-house to represent all the proposed thematic areas of EM (Table 2 in Supplementary material). Whereas existing literature has focused mostly on only one of these dimensions (17, 27–29), we propose them as a theoretical model that displays a full EM-cycle. Furthermore, we link EM with PSC as we believe they are mutually influencing concepts in the context of Safety Management (Figure 1). It is key to understand the types of errors and analyse them to improve the PSC within ICUs (19). The same applies the other way around with PSC influencing the way errors are perceived and handled within an organization.

#### Patient safety culture

PSC was measured using 8 out of 12 dimensions from the German version of the Hospital Survey on Patient Safety Culture (HSOPSC), namely: *Teamwork Within Units*, O*rganizational Learning, Feedback & Communication About Error, Communication Openness, Frequency of Events Reported, Teamwork Across Units, Staffing* and *Non-punitive Response to Error* (30, 31). The remaining 4 dimensions (*Supervisor/ Manager expectations & Actions Promoting Patient Safety, Management Support for Patient Safety, Overall Perceptions of Patient Safety*, and *Handoffs & Transitions*) were excluded as they did not meet the study focus or target group. This is due to the fact that these dimensions were either related specifically to management views of PSC, specific procedures such as handoffs or transitions or in terms of overall perceptions of PSC too vague to draw conclusions for the target group. The questionnaire is a well-established and validated tool, developed by the United States Agency for Healthcare Research and Quality (32). Answers were given on a 5-point Likert scale ranging from 1 = *Strongly disagree/Never* to 5 = *Strongly agree/Always*.

### Statistical analysis

Descriptive data analysis was performed for both EM and PSC using IBM SPSS Statistics, Version 28 (IBM Corp., Armonk, NY, USA). Overall composite percent positive scores of the HSOPSC dimensions were calculated as well as composite scores separated by profession of the participants (nursing or medicine). Therefore, the two highest (*Strongly agree/ Agree* and *Always/ Most of the time*) were combined to calculate agreement (percent positive scores) for each item on each dimension. Negatively worded items were reversely coded.

### Quantitative content analysis

Quantitative Content Analysis (QCA) was performed using f4analyse software v3.3 (audiotranskription.de) for the analysis of EM related data (33). QCA is a technique for systematic evaluation of written or spoken content for example resulting from an interview or as in our case resulting from answers to open-ended questions in a questionnaire. Whenever there is an abundance of content QCA helps to structure communication content systematically, gives an objective insight into data and allows frequency analysis. In contrast to qualitative content analysis, QCA does not aim to interpret single phrases in detail by their meaning but tries to display the overall structure of answers and similarities. Nevertheless, QCA also entails slight qualitative steps to reevaluate the categorical system (34). QCA was performed as follows: First, answers to open-ended questions were briefly examined to propose categorical systems in which answers seem to be optimally clustered. In a second step, a team of three coders matched answers with the suitable category. In this step, responses were interpreted qualitatively. The categorical system was iteratively reevaluated during this process as answers might have depicted a different picture of the data. In addition, subcategories were defined whenever suitable. The category “Unclear/ Other” was used when content did not match any of the defined categories and the mention rate was too low to create a new category or when clear interpretation of the answer was not possible, e. g. due to ambiguity or misspelling. As a result, in the last step, the qualitative data could be interpreted using quantitative measures, such as calculating frequencies, proportions and percentage values.

## Results

### Sample characteristics

654 participants initially opened the survey link and clicked on continue at least once. In total the questionnaire was fully completed by 486 participants, which led to a completion rate of 74%. Partly completed questionnaires were used for analysis as well. Therefore, the study sample varies slightly for each item as participants dropped out of the survey in different subareas.

**Figure 2:**
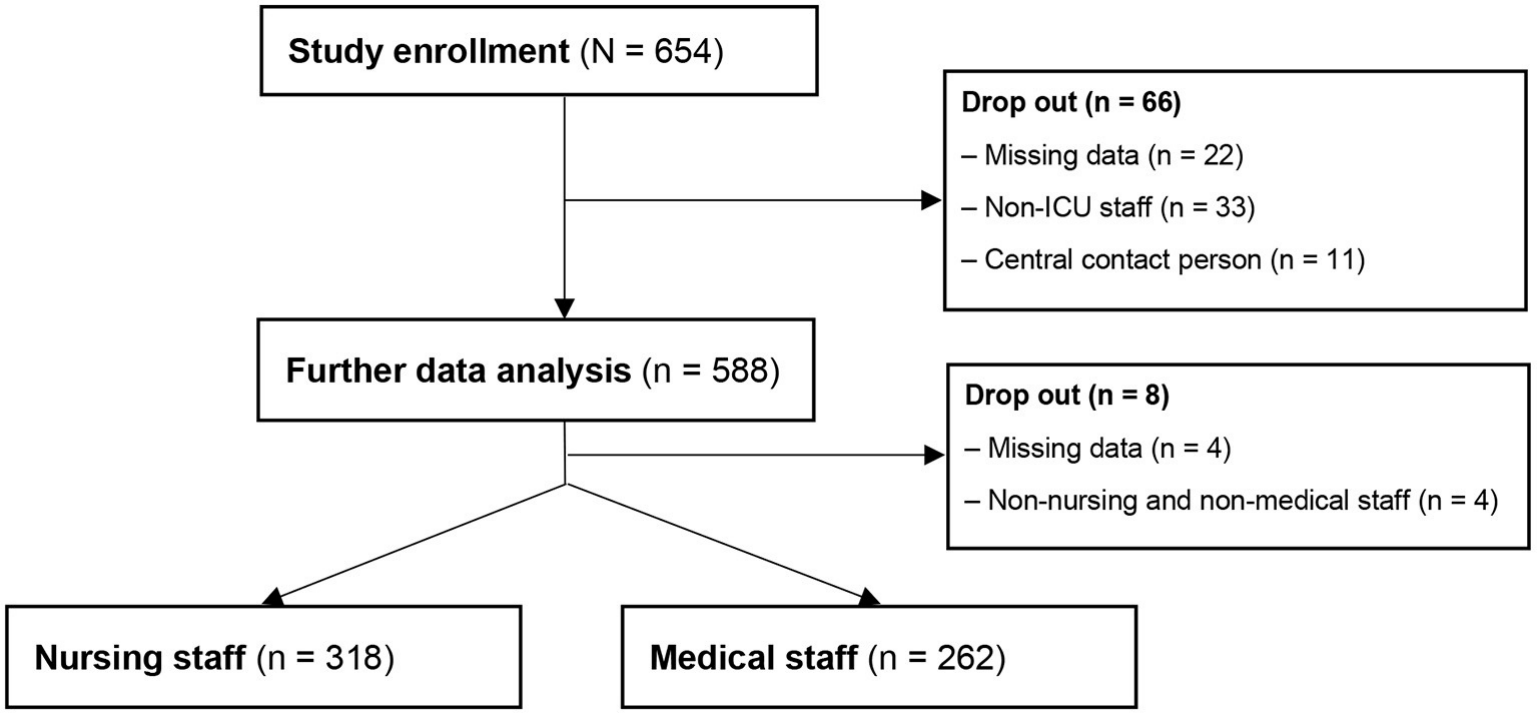
Flow chart.

**Figure 3:**
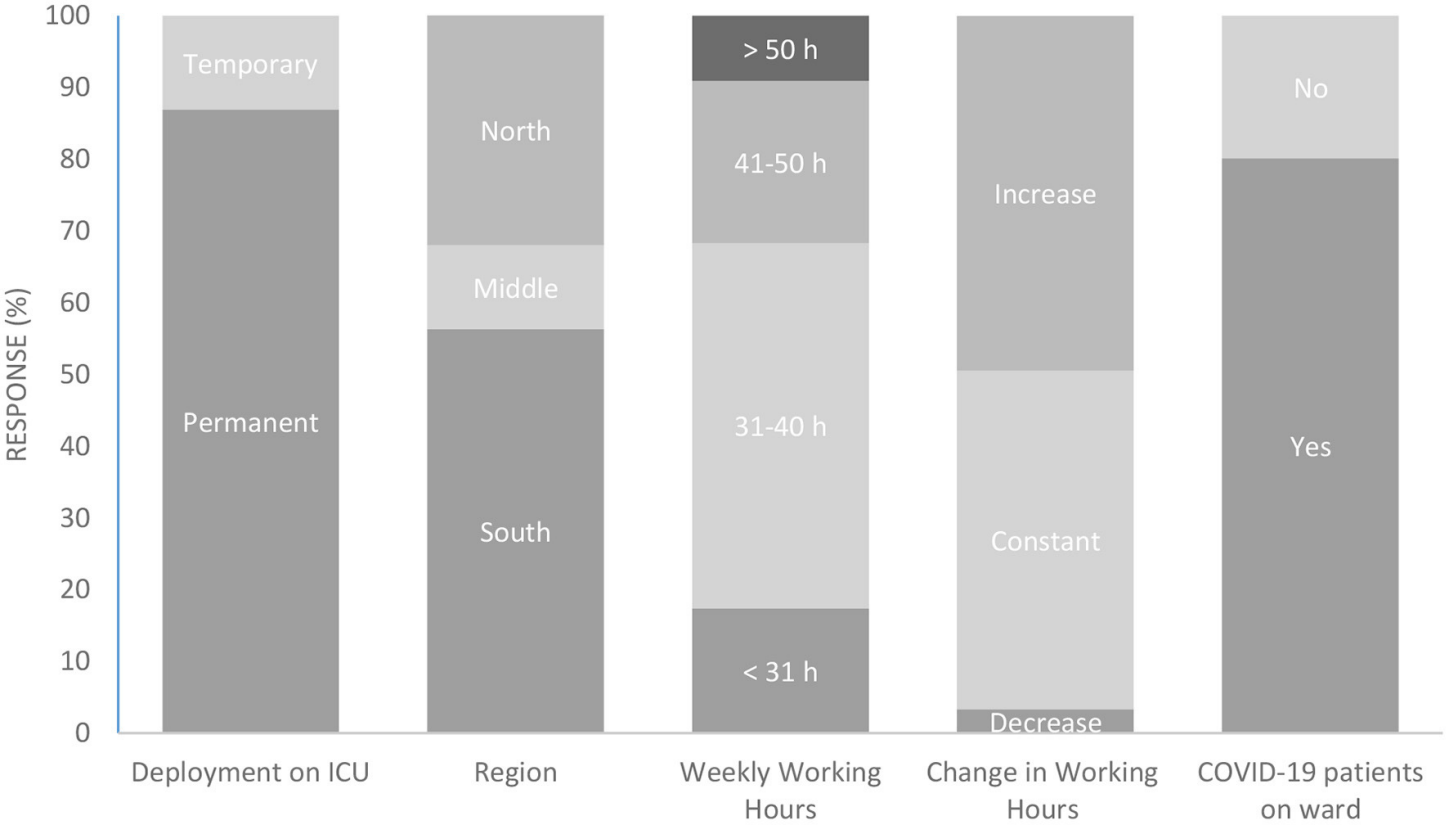
Participant demographics. ICU, Intensive Care Unit; h, hours. Participants were characterized according to the duration of their ICU deployment during the first wave of the COVID-19 pandemic (column 1), the region in Germany where they worked (column 2), their weekly working hours (column 3), and the type of change in working hours during the pandemic (column 4). In addition, the proportion of those who were assigned to a ward with COVID-19 patients is shown (column 5). All response rates are given in percentage terms.

66 participants were excluded from the study sample due to missing data and not fitting the target group of the study. The latter is divided into participants indicating that they were neither deployed nor planned for deployment on ICUS during the first wave of the COVID-19 Pandemic as well as central contact persons that were not part of the target group. The final study sample amounts *n* = 588 ICU staff out of 53 hospitals (Figure 2). Figure 3 displays an overview of demographic characteristics of the study participants while.

**Figure 4:**
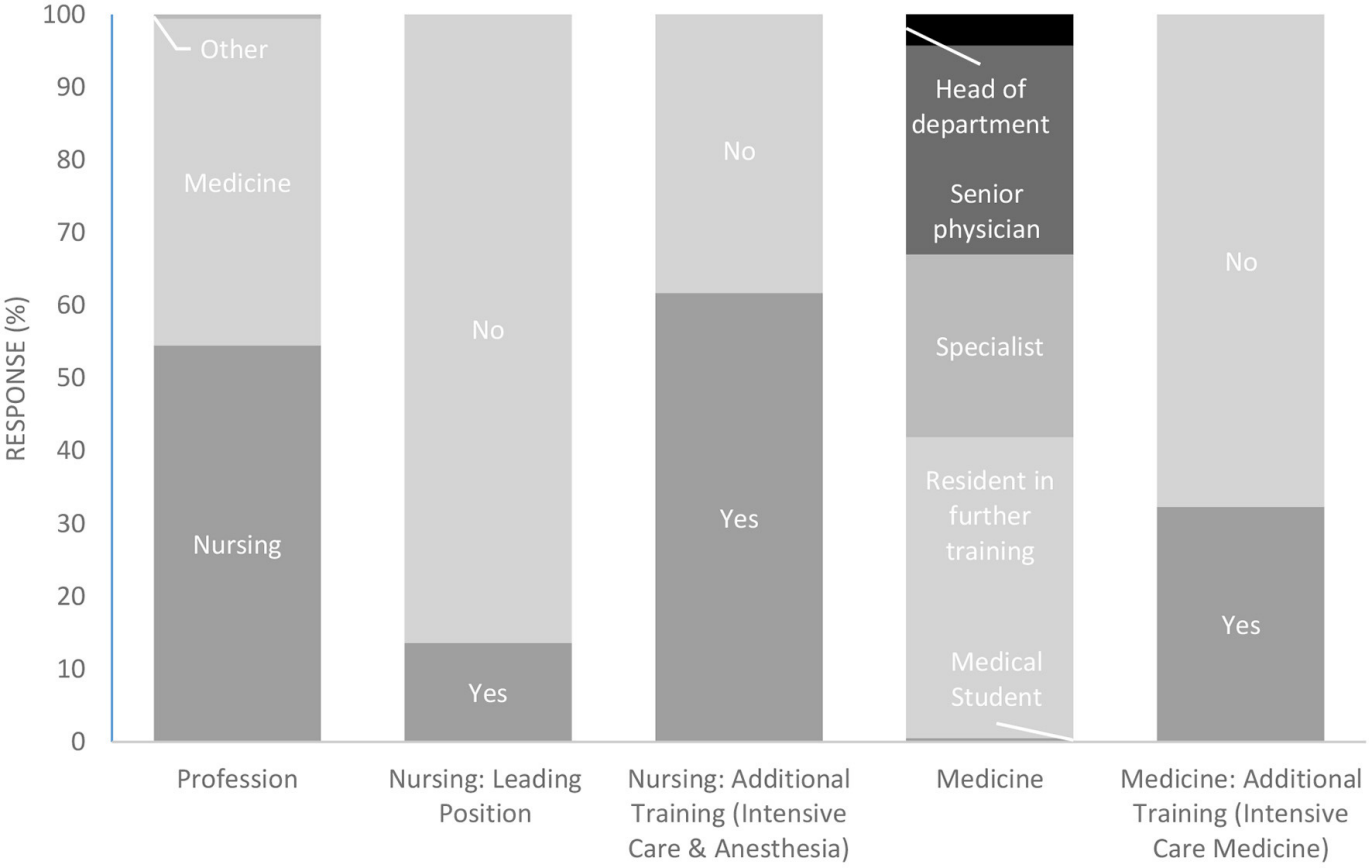
Demographics of medical and nursing staff. Participants were asked about their professional group affiliation (column 1). Nursing staff was asked whether they held a leading position (column 2) and whether they had advanced specialist training in anesthesia and intensive care (column 3). Medical staff was asked about their position (column 4) and whether they had an additional training in intensive care medicine (column 5). Response rates are given in percentage terms for each item.

Figure 4 shows specific demographics of medical and nursing staff regarding position and further training experience.

### Error management analysis

#### Changes in medical errors due to COVID-19 pandemic

60.2% of the participants indicated that medical errors did not increase while 39.8% noticed an increase of errors since the start of the pandemic (*n* = 374). Error increases were noticed mostly in the field of medication errors (17.1%), e. g., administration of the wrong medication or its dosage to a patient. These errors were followed by errors due to lacking human resources (14.5%), e. g., time-delayed medication administration due to insufficient staffing. operator errors while handling respirators (9.4%), e. g. incorrect settings were made or alarms were ignored, were mentioned frequently as well. When asked about a specific error during the first wave of the COVID-19 pandemic, medication errors (25.1%) were mentioned most frequently. These errors were followed by treatment errors (10.3%), e. g., decubiti due to incorrect abdominal positioning of the patient, and operator errors (13.7%), e. g., mixing up of ventilation tubes.

#### Error identification

About every second participant (54.6%) argued that medical errors are identified by colleagues, while 41.7% of the participants stated that they are mostly identified by the person who made the error itself. QCA revealed that the person uncovers these errors through critical incident reporting systems (CIRS), reports to the chief or through communication in team meetings.

#### Error documentation

A large majority of participants (88.8%) stated their department provides a reporting system, but 78.0% of them did not fill out any reporting form since the beginning of the COVID-19 pandemic (*n* = 502). Most of the participants argued that errors were documented in logbooks (97.0%), in-house error reporting systems (88.9%) or by documenting a fictitious patient in the electronic patient record (99.2%), while CIRS (27.7%) or team meetings (36.2%) were least mentioned (*n* = 505).

#### Error communication

61.2% of the participants stated that medical errors are addressed in their department, while this is not the case for 38.8% of the participants due to low quality of error culture (27.8%), supervisors' lack of interest (16.8%), hierarchy (12.7%) and lack of time resources (11.6%). 38.0% of the participants indicated errors are mostly addressed within training courses or within meetings (9.4%) that are held or used for this purpose. Most of the participants (55.4%) experienced monthly, 27.2% annual, 13.9% weekly and 3.5% daily communication about errors (*n* = 287).

#### Error prevention

Most of the participants (55.1%) who noticed an increase in errors during the first wave of the COVID-19 pandemic, did not perceive any prevention actions being implemented. Most frequently mentioned reasons were shortage of personnel resources (20.8%), the medical system in general (14.6%) and low quality of error culture (10.4%). 44.9% of the participants reported that prevention strategies took place and mentioned personnel development (24.6%), control and safety procedures (22.8%) and communication (21.1%) as the most frequent measures. Participants' most frequently reported wishes for future prevention strategies were staff recruitment and deployment planning (27.2%), improvements of working conditions (16.0%) and personnel development (10.1 %).

#### Patient safety culture analysis

Descriptive analysis of the HSOPSC dimensions is depicted in Table 1. *Staffing* received the lowest positive response rates of all dimensions (26.7%) whereas *Teamwork Within Units* (58.0%) was the best rated dimension with a difference of 31.3 percentage points. Overall, both professions evaluated the dimensions in the same direction whereas medical staff tended to rate the situation on ICUs more positively regarding every dimension except for *Communication Openness*. Medical staff rated *Teamwork within Units* (63.2%) while nursing staff rated *Non-punitive Response to Error* (54.4%) most positive.

**Table 1:** Composite percent positive responses of HSOPSC dimensions overall and divided by profession.

**HSOPSC dimensions**	**Composite percent positive response across dimension** ^**a**^		
	**Overall**	**Nursing**	**Medicine**
	**(*n* = 504)**	**(*n* = 275)**	**(*n* = 229)**
Teamwork within units	58.0%	53.8%	63.2%
Organizational learning	42.5%	39.1%	46.6%
Feedback and communication about error	39.2%	37.3%	41.1%
Communication openness	54.2%	54.3%	54.2%
Frequency of events reported	32.3%	32.2%	32.4%
Teamwork across units	31.7%	27.8%	32.4%
Staffing	26.7%	24.5%	29.4%
Non-punitive response to error	57.3%	54.4%	60.8%

^a^Excluding missing responses.

Looking closer into the details of the staffing dimension (Table 2), all the three items concerning workload were rated comparatively low whereas the deployment of temporary staff reaches better positive response marks in both professions.

**Table 2:** Percent positive scores of staffing items overall and divided by profession.

**Staffing items**	**Percent positive response across items** ^**a**^		
	**Overall**	**Nursing**	**Medicine**
	**(*n* = 504)**	**(*n* = 275)**	**(*n* = 229)**
We have enough staff to handle the workload.	15.7%	13.1%	18.9%
Staff in this unit work longer hours than is best for patient care.*	28.2%	29.1%	27.1%
We use more agency/temporary staff than is best for patient care. *	51.6%	47.4%	56.6%
We work in “crisis mode” trying to do too much, too quickly.*	11.3%	8.4%	14.8%

^a^Excluding missing responses; *Item values reverse coded for analysis.

Furthermore, Table 3 displays descriptive differences between hospital types regarding patient safety measures. In almost all dimensions maximum care providers such as university hospitals achieve the lowest results except for the dimensions *Teamwork across Units* (28.0%), *Staffing* (26.9%) and *Non-punitive response to error* (54.8 %).

**Table 3:** Composite percent positive responses of HSOPSC dimensions divided by hospital type.

**HSOPSC dimensions**	**Composite percent positive response across dimension** ^**a**^		
	**Maximum care provider/ University hospital**	**Standard care provider**	**Focal point care provider**
	**(*n* = 273)**	**(*n* = 119)**	**(*n* = 98)**
Teamwork within units	55.7%	60.7%	61.0%
Organizational learning	40.9%	45.8%	41.2%
Feedback and communication about error	37.7%	41.9%	38.8%
Communication openness	51.3%	53.4%	61.6%
Frequency of events reported	30.6%	32.7%	35.5%
Teamwork across units	28.0%	37.3%	26.8%
Staffing	26.9%	24.1%	29.9%
Non-punitive response to error	54.8%	62.5%	41.8%

^a^Excluding missing responses.

Taking a closer look into the scores divided by the region, it becomes visible that the southern part of Germany achieves the best rates in almost all dimensions except for Staffing (28.3%) (Table 4). In this dimension the middle part of Germany scores lower which can be attributed to the individual values of all the items on this dimension (Table 5).

**Table 4:** Composite percent positive responses of HSOPSC dimensions divided by region.

**HSOPSC dimensions**	**Composite percent positive response across dimension** ^**a**^		
	**North**	**Middle**	**South**
	**(*n* = 160)**	**(*n* = 56)**	**(*n* = 275)**
Teamwork within units	53.2%	51.3%	62,0%
Organizational learning	38.9%	39.0%	44,6%
Feedback and communication about error	33.5%	30.4%	43.8%
Communication openness	53.6%	35.4%	59.7%
Frequency of events reported	29.5%	27.1%	34.7%
Teamwork across units	29.1%	26.4%	31.4%
Staffing	33.3%	22.1%	28.3%
Non-punitive response to error	48.2%	50.9%	63.2%

^a^Excluding missing responses.

**Table 5:** Percent positive scores of staffing items divided by region.

**Staffing**	**Composite percent positive response across dimension** ^**a**^		
	**North**	**Middle**	**South**
	**(*n* = 160)**	**(*n* = 56)**	**(n = 276)**
We have enough staff to handle the workload.	11.3%	14.3%	22.5%
Staff in this unit work longer hours than is best for patient care.*	35.6%	25.0%	26.1%
We use more agency/temporary staff than is best for patient care. *	53.1%	43.6%	52.4%
We work in “crisis mode” trying to do too much, too quickly.*	33.1%	5.4%	12.4%

^a^Excluding missing responses; *Item values reverse coded for analysis.

## Discussion

In the present study we argued that EM and PSC are two constructs that operationalize the framework of Safety Management within the healthcare system, specifically on ICUs. Especially in times of crisis it is important to look closely into the distinct aspects of Safety Management and their output on Human Factor. Results from this nationwide status-analysis have uncovered serious potential and a need for structural interventions for improving Safety Management on German ICUs. Despite limited generalizability to other healthcare systems in view of potential social and cultural disparities, we argue that our results care of interest beyond the German healthcare system. This goes in line with the fact that some aspects of critical care are universal among different countries and comparisons can provide useful information (35). Therefore, we believe that international comparisons may provide important insights into how differences in Safety Management translated to failing and succeeding in confronting the pandemic.

Referring to the results on EM, most of the participants did not notice an increase of errors during one of the most intensive phases of the pandemic. This might seem surprising at first but may be related to the fact that adapting to these new circumstances is accompanied by a non-linear pattern of initial acceptable performance level (36). On the other hand, increased workload and pressure could have impaired objective observation of changes in the number of errors. The observed increase in operator errors, specifically related to respirator machines, seems logical as COVID-19 is a virus that primarily affects the respiratory system (37). Thereby it becomes apparent that solely providing the healthcare system with technical devices in an attempt to support or compensate staffing requirements is insufficient. Rather, intensive training is necessary to ensure that medical processes run safely and errors are reduced to a minimum.

Furthermore, errors resulting from staff shortage were mentioned most frequently. Indeed, *Staffing* captures drastic potential for improvement as the worst rated dimension in the analysis of PSC. These results suggest that a lack of personnel can be a source of error which might also be applicable across nations and branches. A recent report by the Agency for Healthcare Research and Quality underlines this hypothesis with similar ratings on this dimension (38). Referring to the system-approach we followed within this study, improvements need to be made but are not limited to working conditions. With a current tendency that the shortage of skilled professionals in the medical system will be ongoing, systemic factors might even be the main reasons for errors while individual failures happen as consequence. If so, prevention strategies need to focus primarily on the system and the working conditions. In fact, PSC analysis showed that the deployment of temporary staff can be one option to improve the tense condition of staff shortage, but further and sustainable measures are needed to reduce the overall workload and create serious improvements that impact PSC. Beyond that, actions are also needed regarding the qualification of existing staff as this was one of the most mentioned strategies wished for to prevent future errors from happening.

Furthermore, the results on Error Identification shed a light on how we currently record medical errors. While the majority of errors were identified either by the person oneself or colleagues and almost all the participants stated their department provides CIRS, only few participants reported to have actively used existing reporting systems during the first wave of the COVID-19 pandemic. This is not surprising as research has already shown that it is the “human nature not to report errors” (16). Nevertheless, results show that existing efforts might have fallen too short and we need more effective ways of reporting errors to depict a realistic overview—especially in times of crisis.

In addition, with the documentation of reported errors mostly conducted through logbooks or in-house reporting systems but a high amount of people stating that these errors were not addressed, a major loss of information is conceivable. When only a few errors of an already incomplete overview of errors are addressed, potential for learning is limited. This also becomes clear when looking at a lack of interest by authorities and a low quality of error culture as reported reasons for not addressing errors. This highlights not only the importance of effective communication (16) but also addresses a need for intensified efforts in error culture. Hence, all EM dimensions have the potential to influence individual and organizational learning processes because learning from errors goes in line with preventing severe errors from happening to build individual as well as organizational resilience (20).

The results of a study prior to pandemic times by Gambashidze et al. (39) draw a similar picture of the patient safety dimensions that is relatable to our findings and underlines the importance of improving patient safety (39). The results lead to the assumption, that Safety Management has not improved greatly in the years leading up to the pandemic or the crisis has damped past efforts. Interestingly, most of the dimensions that are specifically related to errors were rated worse by our study sample which might be caused by the crisis situation. Nevertheless, the comparison of these results needs to be seen in light of some limitations as we included hospitals all over Germany which were not limited to university hospitals and our study focused on intensive care units.

In conclusion, the present status-analysis of Safety Management in times of crisis revealed some serious potential for improvement on German ICUs, especially in an international comparison (40). It outlined the necessity to not only make Safety Management and its facets an ongoing commitment throughout healthcare organizations (26) but also, to intensify and especially tailor these Safety Management measures. One way to do so might be to further integrate simulation training with EM instructions into personnel development (41). Since our results have shown that communication is a critical aspect when it comes to sufficient error reporting and addressing them in a respectful manner, we believe that intensified teaching of communication strategies, such as closed-loop communication, can be beneficial for staff development and Safety Management in general. Other ways might also include structured approaches, e. g. the concept of Circle Up, to improve communication and information flow within ICU teams (42).

## Limitations

To our knowledge, this is the first study which assesses the status of Safety Management in times of crisis within the scope of a nationwide survey on German ICUs. Nevertheless, the findings of this study need to be seen in light of some limitations. First, the present status-analysis is based on a data collection at one point of time. Given the knowledge that PSC and EM are dynamic processes requiring ongoing efforts (19), it seems important to examine the development over a longer period, for example how PSC and EM evolve looking at the transition of the pandemic into an endemic state or in post-pandemic times. Furthermore, we are aware that there are relevant questions that cannot be answered by the investigation of one time point—such as differences between profession groups or regions. With our work we hope to stimulate further research activities which are hypothesis-driven and can answer these questions with sufficient certainty. Second, the current study does not claim to be exhaustive regarding subjective error reporting. As known from previous research, it is difficult to measure the occurrence and severity of errors objectively as it relies on the subjective willingness to contribute to error reporting and as it is the “human nature not to report errors” (16). A combination of qualitative and quantitative as well as subjective and objective error reportings might be helpful to extend insights. Lastly, the findings of our study should be interpreted tailored to German ICUs. The generalization to the wider population of critical care staff in Germany and beyond the German healthcare system is limited. Nevertheless, it can be assumed that results might be applicable to an international and cross-sectoral context. Future studies on Safety Management should explore the transferability of these results and test this assumption across different nations and industries. Finally, as the findings of this study are based on staff perceptions, future studies may additionally include results from CIRS to avoid overrepresentation of participants, who might have been more likely to respond to the study invitation because of strong views or particular experiences. Thus, the non-response bias should be taken into account when interpreting the study results. When participating in a survey, it is most likely that people with experiences at the far end of a scale make up a big part of the study sample whereas a great amount of people with moderate experience tend to not respond or participate in the study. This might be one reason for an unknown amount of potential study participants with moderate experiences that decided to not participate in our study. Another reason for non-response could be that reporting medical errors is a sensitive issue which may have been an obstacle for participation. Furthermore, a lack of time and personnel resources due to the pandemic might be a reason for non-response. These potential reasons may have caused non-response and eventually biased the results.

## Conclusion

Human factor in High Reliability Organizations such as healthcare institutions is an everlasting concern in terms of safety culture as well as the assessment of errors. The present status-analysis highlights this importance but also urges the significance of improving systemic factors such as working conditions and staffing that need to be considered when interpreting Safety Management in the healthcare system.

## Data availability statement

Study material and datasets are available from the corresponding author upon reasonable request.

## Ethics statement

The studies involving human participants were reviewed and approved by the Ethics Review Board of the University Hospital RWTH Aachen (Ethics Review Board document 459/20) on 01.12.2020. Written informed consent for participation was not required for this study in accordance with the National Legislation and the Institutional Requirements.

## Author contributions

MS made substantial contributions regarding study conceptualization and design, conducted the data analysis, and wrote the manuscript. SL and MK were involved in the analysis and interpretation of the data. SL, MK, ML, FJ, LT, and GH reviewed and made substantial contributions to the manuscript. SS critically reviewed the manuscript for important intellectual content, supervised the study and supported MS as senior investigator. All authors contributed to the conceptualization and design of this study, including the preparation of study material, and reviewed and revised the manuscript. All authors agree to be accountable for all aspects of this work and approve the final manuscript as submitted.

## Funding

The study was partly supported by the Federal Ministry of Education and Research of the German Federal Government (Project egePan Unimed, Grant nr. 01KX2021) and by in-house funding of the project partners.

## Conflict of interest

ML and BS declare that they received research grants from the German Federal Ministry of Education and Research (BMBF) and from Erasmus+. ML declares the immediate past presidency of the Society of Simulation in Europe (SESAM). BS declares the deployment as a trainer for medical simulation training at CATHI GmbH and a trainer for medical emergency training at Polyclinic for Dental Preservation and Periodontology and a received payment for expert testimony from M3i GmbH. The remaining authors declare that the research was conducted in the absence of any commercial or financial relationships that could be construed as a potential conflict of interest.

## Publisher's note

All claims expressed in this article are solely those of the authors and do not necessarily represent those of their affiliated organizations, or those of the publisher, the editors and the reviewers. Any product that may be evaluated in this article, or claim that may be made by its manufacturer, is not guaranteed or endorsed by the publisher.

## Abbreviations

CIRS: Critical Incident Reporting Systems
CMO, Chief Medical Officer EM: Error Management
HSOPSC: Health Survey on Patient Safety Culture
ICUs: Intensive Care Units
PAE: Preventable Adverse Events
PSC: Patient Safety Culture
QCA: Quantitative Content Analysis.
