# Supplementary material for: Safety management in times of crisis: Lessons learned from a nationwide status-analysis on German intensive care units during the COVID-19 pandemic
Source: Front Med (Lausanne). 2022 Oct 5;9:988746. doi: 10.3389/fmed.2022.988746 (PMC9583873; doi:10.3389/fmed.2022.988746)
Supplement: Supplementary file 2 [file Table_2.pdf]

# Error Management Items

| Nr | Dimension | Original German Version | English Translation |
|---|---|---|---|
| 1 | **COVID-19** | Beschreiben Sie bitte kurz (stichwortartig) einen ausgewählten Fehler, der im Zeitraum seit Beginn der COVID-19-Pandemie in Ihrer Abteilung/ Klinik passiert ist. (Hierbei ist es unwichtig, ob dieser Fehler Ihnen oder jemand anderem passiert ist.) | Please briefly describe (in key words) a selected error that has occurred in your department/ clinic in the period since the COVID 19 pandemic began. (It does not matter whether this error happened to you or someone else). |
| 2 | | Welche Fehler sind seit Beginn der COVID-19 Pandemie ihrer Meinung nach stärker aufgetreten? | In your opinion, which errors occurred more frequently since the start of the COVID-19 pandemic? |
| 3 | **Error Identification** | Durch wen werden in Ihrer Abteilung/ Klinik kritische Ereignisse und Fehler hauptsächlich aufgedeckt? | By whom are critical events and errors mainly detected in your department/ clinic? |
| 4 | | Wie decken die Personen den eigens verschuldeten Fehler meist auf? | How do individuals usually uncover the self-inflicted error? |
| 5 | **Error Documentation** | Wie werden Fehler berichtet/ dokumentiert? | How are errors reported/documented? |
| 6 | **Error Communication** | Wird Fehlermanagement in Ihrer Abteilung/ Klinik thematisiert? | Is error management addressed in your department/clinic? |
| 7 | | Wie wird Fehlermanagement in Ihrer Abteilung/ Klinik thematisiert? | How is error management addressed in your department/clinic? |
| 8 | | Warum wird Ihrer Meinung nach Fehlermanagement in Ihrer Klinik/ Abteilung nicht thematisiert? | In your opinion, why is error management not addressed in your clinic/ department? |
| 9 | | Wie oft wird Fehlermanagement in Ihrer Abteilung/ Klinik thematisiert? | How often is error management addressed in your department/clinic? |
| 10 | **Error Prevention** | Wurden bereits Maßnahmen ergriffen, um diese Fehler zu vermeiden? | Are there already actions been taken to avoid these errors? |
| 11 | | Welche Maßnahmen wurden ergriffen, um diese Fehler zu vermeiden? | What actions have been taken to avoid these errors? |
| 12 | | Warum wurden Ihrer Meinung nach keine Maßnahmen ergriffen, um diese Fehler zu vermeiden? | Why do you think no actions have been taken to avoid these errors? |
